# Impact of the COVID-19 Pandemic on Prenatal Care Utilization Among Italian and Immigrant Pregnant Women: A Multicenter Survey

**DOI:** 10.3389/ijph.2024.1606289

**Published:** 2024-02-19

**Authors:** Luz Maria Vilca, Laura Sarno, Davide Passoni, Patrizio Antonazzo, Edda Pellegrini, Maurizio Guida, Elena Cesari, Irene Cetin

**Affiliations:** ^1^ Department of Woman, Mother and Neonate, Buzzi Children’s Hospital, University of Milan, Milan, Italy; ^2^ Chickahominy Health District, Virginia Department of Health, Ashland, VA, United States; ^3^ Department of Neurosciences, Reproductive Science and Dentistry, University of Naples Federico II, Naples, Italy; ^4^ Unit of Obstetrics and Gynecology, Maurizio Bufalini Hospital, AUSL Romagna, Cesena, Italy; ^5^ Maternal and Child Committee-Lombardy Region, Milan, Italy

**Keywords:** COVID-19 pandemic, immigrant, prenatal care services, maternal immunization, ultrasound, emergency, care, prenatal course

## Abstract

**Objectives:** To compare the utilization of prenatal services between immigrant and Italian women during the COVID-19 pandemic.

**Methods:** A cross-sectional survey was conducted at 3 maternity care centers in Italy.

**Results:** We included 1,312 women, 1,198 (91.3%) were Italian and 114 (8.7%) were immigrants. A significantly higher proportion of Italians underwent 8 or more prenatal care visits (64.4% vs. 54.4%, *p* = 0.03) and more immigrants than Italians attended their appointments at hospital settings (45% vs. 18%, *p* < 0.001). Regarding prenatal course, Italians were more likely than immigrants to attend a non-hospital setting or an online class (49.6% and 30.2% vs. 34.9% and 11.6%, *p* = 0.008). A higher influenza vaccine uptake among immigrants compared with Italians was observed (39.5% vs. 19.8%, *p* < 0.001). Among women not receiving certain prenatal services, immigrants were more likely to state COVID-19 pandemic was the main reason for non-compliance.

**Conclusion:** Immigrant pregnant women were more likely to receive prenatal services at a hospital setting than their Italian counterparts. Among women who did not comply with prenatal services, immigrants were more likely to cite the pandemic as their main reason.

## Introduction

After the declaration of the COVID-19 pandemic on 11 March 2020 [1], Italy rapidly became the European country most affected by the spread of the SARS-CoV-2 transmission and in March–April 2020 over 28,000 deaths were reported in this country [2]. During the first pandemic wave (from February to May 2020) a national lockdown was established as well as several containment measures such as quarantine, schools’ closure, cancellation of events, restrictions on mass gatherings in public and private places, among others, to decelerate the diffusion of COVID-19 pandemic in society. These measures started on 8 March 2020, and ended on 18 May 2020 [3].

This was followed by a transition period from June to mid-September 2020 in which some measures were removed, but a strong contact tracing system was implemented. The second peak started in mid-September and in October 2020 the virus started spreading faster, new restrictions were adopted and a differentiation of containment measures according to the risk of diffusion was implemented [4, 5].

The surges in COVID-19 cases severely stressed healthcare systems and public health infrastructure and some facilities were forced to adopt crisis standards of care to address the needs of larger numbers of patients [6]. With regards to maternal and child services, Lazzerini et al. reported a reduction in pediatric emergency department visits ranging from 73% to 88% during the first national lockdown (March 1st, 2020 to March 27th, 2020) compared to the same period in 2019 and 2018 [7]. Similarly, Cena et al. reported that during the first wave only 28% of surveyed maternal and perinatal healthcare facilities continued to provide outpatient routine visits and examinations as usual, while 59% of them provided visits to a limited extent. In contrast, the majority of hospitals were available for emergency visits [8]. This negative impact on the functioning of maternal healthcare services coupled with the reticence among pregnant women to avoid potential exposure to SARS-CoV-2 might have caused not only a significant reduction in access to prenatal services but also worse maternal health outcomes.

Pregnant population is particularly vulnerable to altered or delayed maternal care and this could be even more critical for certain subpopulations with historically lower healthcare access and worse maternal health outcomes, such as immigrant pregnant women who represented 21% of births in Italy in 2020, surpassing 30% of births in certain regions such as Emilia-Romagna, Lombardy, Liguria and Marche [9].

In United Kingdom, Vousden et al. have confirmed that pregnant women hospitalized with SARS-CoV-2 were more likely to be Black, Asian or of other minority ethnicity, irrespective of symptom status, age, body mass index and medical comorbidities [10]. The disparities in maternal outcomes and less or delayed access to prenatal care services between immigrant and autochthonous pregnant women have been described before the pandemic in Italy [11]. Since COVID-19 has widened the health inequity gap among ethnic minorities, the aim of our study was to assess the differences in access to selected antenatal care (ANC) services among immigrant versus Italian women from May to December 2020.

## Methods

### Study Design and Population

A cross-sectional study was conducted between May and December 2020 in the maternity care units (MCU) of three tertiary care hospitals: *Buzzi Children’s University Hospita*l, Milan (Northern Italy), *Maurizio Bufalini Hospital*, Cesena (Central Italy) and Federico II University Hospital, Naples (Southern Italy). During 2020 these MCU attended 3,170, 1,803 and 2,723 births, respectively, in Italian cities with diverse immigration flows.

A convenience sample of pregnant women who satisfied the eligibility criteria was recruited at postnatal wards at each MCU. The inclusion criteria includes: a) being ≥18 years old, b) having a live birth between 1 May 2020, and 31 December 2020, and c) provision of informed consent. All eligible women were approached during their hospital stay and invited to participate by one member of the research team and after giving their consent, the survey was administered in a quiet room. The survey was available in four different languages: Italian, English, Arabic and Mandarin Chinese and assistance in filling in the survey was offered, if requested by the women. In this study, an immigrant woman was defined as any participant who was not born in Italy and stated that their current residency status was Italy.

### Survey Questionnaire

The research team developed an anonymous, self-completed and structured questionnaire including 32 questions that could be completed in about 10 min. No financial compensation or incentives were offered to participants and participation was voluntary.

The survey included demographic and clinical questions such as age, country of origin, education level (low, middle, or high), civil status (single, cohabiting or married), employment status (employed, housewife or unemployed), parity (primiparous, multiparous) and pregnancy complications (yes/no). We also asked about attendance or receipt of the most common prenatal care services/prenatal tests offered in Italy: number of ANC appointments received, number and time of the ultrasound scans performed during pregnancy, prenatal course attendance, influenza and pertussis vaccination receipt; obstetric emergency room visit(s), oral glucose tolerance test and Group B *Streptococcus* (GBS screening). In the case the women did not attend or receive those services/tests, we also collected information about the reasons for non-compliance.

### Data Analysis

The results are presented as proportions of respondents to individual questions, excluding nonresponses from the denominators. Mean and standard deviation or median and interquartile range were estimated for continuous variables. Chi square or Fisher’s exact tests were applied to compare differences in demographic variables, compliance with seven prenatal care services, reasons for non-compliance and preferred setting for prenatal care services among Italian and immigrant women. Two-tailed were applied and findings were reported as significant at *p* < 0.05.

Factors associated with compliance with selected 5 ANC services were analyzed using binary logistic regression analysis. Collinearity and interactions between variables were checked. A backward stepwise logistic regression was used to identify possible predictors of compliance with each one of the 5 ANC services selected (8 or more ANC visits, emergency room utilization, prenatal course attendance, influenza and pertussis vaccination). We started with a full or upper model which includes all the potential factors associated with ANC services compliance. And, at each step, variables were removed based on *p*-values, and the *p*-value threshold of <0.1 was used to set a limit on the total number of variables included in the final models. The lower model includes variables that were considered relevant by subject-matter knowledge such as age, immigrant status, education level, civil status and parity.

Results of these analyses are presented as odds ratios (OR) and 95% confidence intervals (95% CI). The statistical package R Core Team (2019) was used (“R: A language and environment for statistical computing. R Foundation for Statistical Computing, Vienna, Austria,” n.d.).

This study was approved by the local Ethical Committee (“*Comitato Etico Milano Area 1*”) with the reference number 0056034.

## Results

From May to December 2020, 1,381 surveys were collected. Sixty-nine surveys were excluded because 40 (58.0%) were not fully completed, 17 (24.6%) provided inconsistent responses (i.e., women who declared to attend or receive a service/test and then incorrectly answered subsequent questions about the reason for non-compliance) and 12 (17.4%) were both, not fully completed or with inconsistent responses. Thus, the total study sample included 1,312 respondents: 344 (26.2%) were from Milan; 357 (27.2%), from Cesena; and 611 (46.6%), from Naples. Socio-demographic and clinical characteristics of the total study participants and by citizenship are reported in Table 1. The number of Italian participants was 1,198 (91.3%) and the number of immigrant women was 114 (8.7%). About 19% (64 out of 344) study participants in the Milan study center were immigrant women; 11% (39 out of 357), in Cesena and 9.7% (11 out of 611), in Naples. Country of origin of immigrants enrolled by study center and socio-demographic and clinical characteristics by study center are shown in Supplementary Tables S1, S2, respectively. The mean maternal age was 32.7 ± 5.0 years for Italian women and 31.6 ± 5.3 years for immigrant women. The level of education was significantly lower among immigrants compared to Italians (35.1% immigrant vs. 47% Italian women with high education level; *p* = 0.03). Also, a lower proportion of immigrant than Italian women stated they were currently working (54.4% vs. 64.3%, respectively; *p* = 0.04) (Table 1).

**TABLE 1:** Characteristics of surveyed pregnant women. Italy, 2020.

Socio-demographic and clinical characteristics	Total survey respondents	Italian women	Immigrant women	*p*-value
	*N* = 1312	*N* = 1198 (91.3%)	*N* = 114 (8.7%)	
Study center				**<0.001**
Naples	611 (46.6)	600 (50.1)	11 (9.7)	
Milan	344 (26.2)	280 (23.4)	64 (56.1)	
Cesena	357 (27.2)	318 (26.5)	39 (34.2)	
Age group (years)				0.09
<25	71 (5.4)	62 (5.2)	9 (7.9)	
25–35	757 (57.7)	684 (57.1)	73 (64.0)	
>35	484 (36.9)	452 (37.7)	32 (28.1)	
Marital status				0.53
Married	796 (60.7)	730 (60.9)	66 (57.9)	
Single/Cohabiting	516 (39.3)	468 (39.1)	48 (42.1)	
Education level^a^				**0.03**
Low	185 (14.1)	162 (13.5)	23 (20.2)	
Middle	524 (39.9)	297 (39.5)	51 (44.7)	
High	603 (46.0)	742 (47.0)	40 (35.1)	
Work status				**0.04**
Employed	832 (63.4)	770 (64.3)	62 (54.4)	
Housewife/Unemployed	480 (36.6)	428 (35.7)	52 (45.6)	
Current pregnancy				1.00
Singleton	1,285 (97.9)	1,173 (97.9)	112 (98.3)	
Twins	27 (2.1)	25 (2.1)	2 (1.7)	
Parity				0.27
Primiparous	675 (51.4)	622 (51.9)	53 (46.5)	
Multiparous	637 (48.6)	576 (48.1)	61 (53.5)	
Number of children				0.007
None	675 (51.4)	62 (51.9)	53 (46.5)	
1	489 (37.3)	451 (37.7)	38 (33.3)	
2 or more	148 (11.3)	125 (10.4)	23 (20.2)	
Number of antenatal care visits				**0.03**
<8 visits	479 (36.5)	427 (35.6)	52 (45.6)	
8 or more visits	833 (63.5)	771 (64.4)	62 (54.4)	
Pregnancy complications				0.77
No	1,128 (86.0)	1,031 (86.1)	97 (85.1)	
Yes	184 (14.0)	167 (13.9)	17 (14.9)	
Quarantine during current pregnancy				0.50
No	1,164 (88.7)	1,065 (88.9)	99 (86.8)	
Yes	148 (11.3)	133 (11.1)	15 (13.2)	
Positive COVID test during current pregnancy?				0.78
No	1,271 (96.9)	1,161 (96.9)	110 (96.5)	
Yes	41 (3.1)	37 (3.1)	4 (3.5)	

^a^ Lower education = no secondary school diploma; Middle education = completed secondary school with diploma; Higher education = continued education beyond secondary school.

Bold values denote *p*-values < 0.05.

**FIGURE 1:**
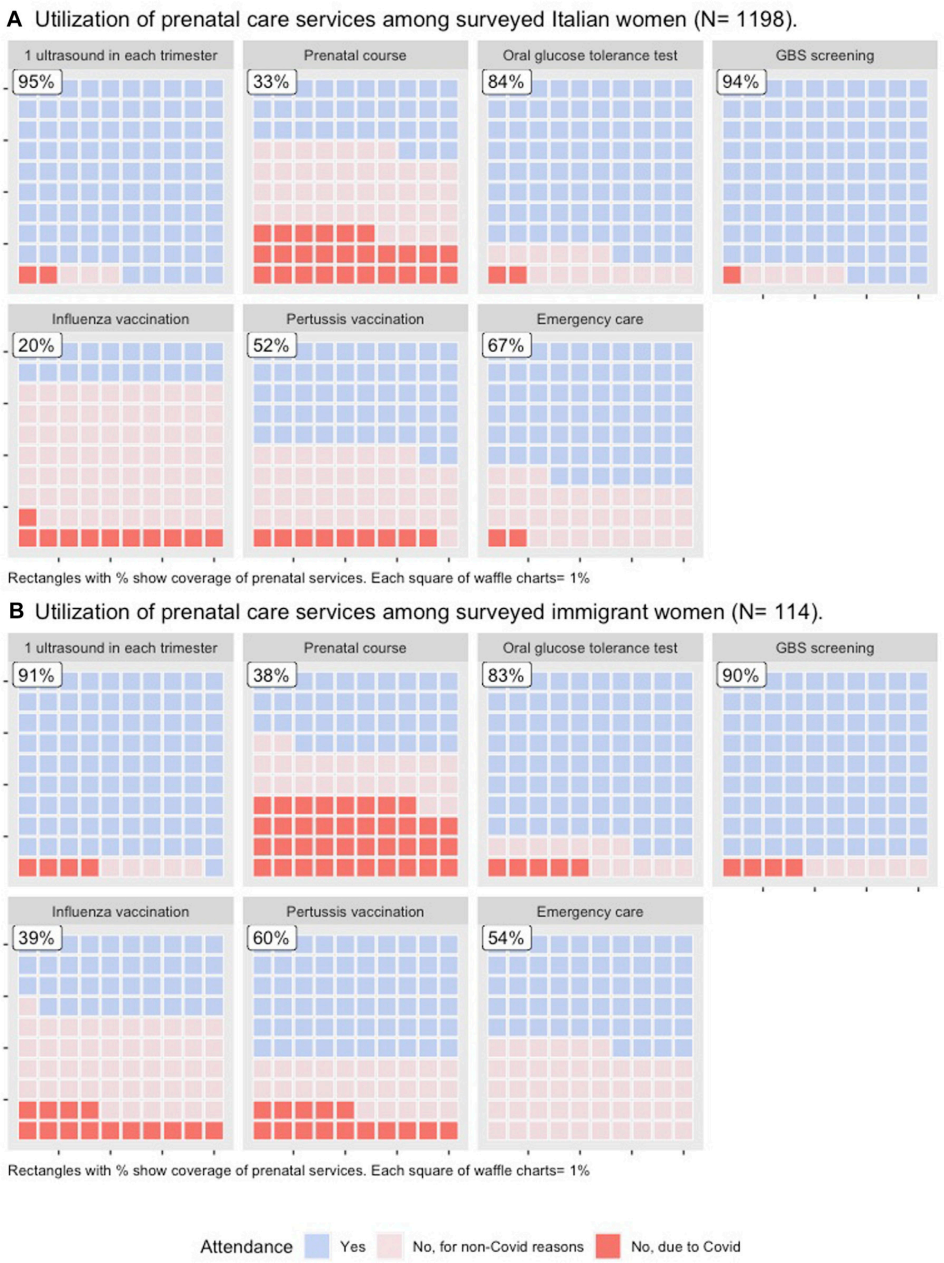
Utilization of seven prenatal care services among Italian and immigrant women. Italy, 2020.

Compliance and non-compliance for seven prenatal services assessed in the survey according to citizenship status, as well as the reasons for non-compliance are shown in Figure 1 and Table 2.

**TABLE 2:** Prenatal care services utilization and reasons for non-compliance among Italian and immigrant women. Italy, 2020.

A. Prenatal care services’ compliance by citizenship and respective *p*-values.			
Prenatal care services	Citizenship		*p*-value
	Italian	Immigrant	
	*N* = 1,198	*N* = 114	
Number of prenatal visits			**0.03**
<8 visits	427 (35.6%)	52 (45.6%)	
≥8 visits	771 (64.4%)	62 (54.4%)	
At least 1 ultrasound in each trimester			0.12
No	63 (5.26%)	10 (8.8%)	
Yes	1,135 (94.7%)	104 (91.2%)	
Glucose tolerance test			0.77
No	187 (15.6%)	19 (16.7%)	
Yes	1,011 (84.4%)	95 (83.3%)	
GBS screening			0.12
No	71 (5.9%)	11 (9.6%)	
Yes	1,127 (94.1%)	103 (90.4%)	
Emergency care visit			**0.005**
No	391 (32.6%)	52 (45.6%)	
Yes	807 (67.4%)	62 (54.4%)	
Prenatal course			0.32
No	801 (66.9%)	71 (62.3%)	
Yes	397 (33.1%)	43 (37.7%)	
Influenza vaccination			**<0.0001**
No	961 (80.2%)	69 (60.5%)	
Yes	237 (19.8%)	45 (39.5%)	
Pertussis vaccination			0.10
No	580 (48.4%)	46 (40.3%)	
Yes	618 (51.6%)	68 (59.7%)	

B. Reasons for not attending prenatal care services by citizenship and respective *p*-values.			
Reasons for non-compliance with prenatal care services	Citizenship		*p*-value
	Italian	Immigrant	
At least 1 ultrasound in each trimester			1.00
No, due to COVID	29/63 (46.0%)	5/10 (50%)	
No, due to other reason	34/63 (54.0%)	5/10 (50%)	
Glucose tolerance test			0.16
No, due to COVID	25/187 (13.4%)	5/19 (26.3%)	
No, due to other reason	162/187 (86.6%)	14/19 (73.7%)	
GBS screening			0.05
No, due to COVID	8/71 (11.3%)	4/11 (36.4%)	
No, due to other reason	63/71 (88.7%)	7/11 (63.6%)	
Emergency care visit			0.09
No, due to COVID	22/391 (5.6%)	0/52 (0%)	
No, due to other reason	369/391 (94.4%)	52/52 (100%)	
Prenatal course			**<0.001**
No, due to COVID	313/801 (39.1%)	43/71 (60.6%)	
No, due to other reason	488/801 (60.9%)	28/71 (39.4%)	
Influenza vaccination			**0.03**
No, due to COVID	130/961 (13.5%)	16/69 (23.2%)	
No, due to other reason	831/961 (86.5%)	53/69 (76.8%)	
Pertussis vaccination			**0.003**
No, due to COVID	108/580 (18.6%)	17/46 (37%)	
No, due to other reason	472/580 (81.4%)	29/46 (63%)	

Bold values denote *p*-values < 0.05.

The factors associated with 5 selected prenatal care services (8 or more ANC visits, emergency room visit, prenatal course attendance, influenza and pertussis vaccine receipt) among the whole study population are shown in Supplementary Tables S3, S4 (unadjusted and adjusted OR, respectively).

### Number of ANC Visits

The mean number of prenatal visits among Italian women was 8.2 ± 2.8 vs. 8.0 ± 3.3 among immigrant women (*p* = 0.47). When we asked participants if they had attended all ANC visits recommended, almost all Italians and immigrants (95.6% and 96.7%, respectively) stated they did. However, among those who did attend all their prenatal care appointments, a higher proportion of immigrant women stated they visited a hospital setting for doing so compared to Italian women (45% vs. 18%, *p* < 0.0001).

A higher proportion of Italians compared to immigrants attended 8 or more visits (64.4% vs. 54.4%, respectively; *p* = 0.03).

In the whole study population, being from Naples (adjusted odds ratio (aOR) = 2.03; 95% CI, 1.56–2.65), and having any pregnancy complication (aOR = 1.97; 95% CI: 1.38–2.85) were significantly associated with having 8 or more visits. In contrast, women who reported being a housewife or unemployed were less likely to have 8 or more ANC visits (aOR = 0.65; 95% CI, 0.49–0.86) (Supplementary Table S4).

### At Least One Ultrasound in Each Gestational Trimester, Glucose Tolerance Test and GBS Screening

Non-statistically significant differences between Italian and immigrant were identified for the following services: at least 1 ultrasound in each gestational trimester (94.7% vs. 91.2%, respectively; *p* = 0.12); glucose tolerance test (84.3% vs. 83.3%, respectively; *p* = 0.77); and GBS screening (94.1% vs. 90.4%, respectively; *p* = 0.12). However, the proportion of Italians performing these services was slightly higher compared to immigrants.

Among women performing at least 1 scan by trimester, a significantly higher proportion of Italians compared to Immigrant women (76.1% vs. 52.9%, *p* < 0.0001) mentioned they visited a non-hospital setting to perform this service.

Among women who did not perform a GBS screening, a higher percentage of immigrants than Italians stated that the reason was the COVID-19 pandemic (36.4% vs. 11.3%, *p* = 0.05). Similarly, a higher proportion of immigrants than Italian respondents cited the pandemic as the reason for not complying with the oral glucose test, even if the difference was not statistically significant (26.3% vs. 13.4%; *p* = 0.24).

### Emergency Room Visit

Italian women reported more frequently at least one visit to the emergency department than immigrant women (67.4% vs. 54.4%, respectively; *p* = 0.005). Interestingly, among immigrant women who did not receive obstetrical emergency care, none of them mentioned the COVID-19 pandemic as the main reason, and only 5.6% of Italian women, who did not attend Emergency care, stated that the main reason for non-attendance was the pandemic (Table 2).

Among the study population, women enrolled in Naples received emergency care 3 times more frequently than patients from Cesena and Milan (aOR = 3.40; 95% CI, 2.57–4.54). Also, multiparous women were less likely to visit the Emergency Room compared with primiparous (aOR = 0.66; 95% CI, 0.51–0.86) (Supplementary Table S4).

### Prenatal Course Attendance

No differences in compliance were observed between Italian and immigrant women (37.7% vs. 33.1%, *p* = 0.32). Nonetheless, among immigrant women who did attend a prenatal course, the setting more frequently used was the hospital followed by non-hospital settings and the online version of the course (53.3%, 34.9% and 11.6%, respectively). In contrast, among Italian women who did attend the prenatal course, the non-hospital setting was cited more frequently than the online version of the course or the hospital setting (49.6%, 30.2% and 20.2%, respectively). The differences in the prenatal course settings used by Italian and immigrant women were statistically significant (*p* = 0.008).

Among women who did not attend a prenatal course, a significantly higher proportion of Immigrants compared to Italian women stated that the main reason was the COVID-19 pandemic, (60.6% vs. 39.1%, respectively; *p* < 0.001) (Table 2).

Among all survey respondents, being enrolled at the Naples site (aOR = 0.24; 95% CI, 0.17–0.32), having a medium or low educational level (aOR = 0.48; 95% CI, 0.35–0.65 or aOR = 0.45; 95% CI, 0.27–0.73), being housewives/unemployed (aOR = 0.65; 95% CI, 0.47–0.89), and being multiparous (aOR = 0.17; IC 95%, 0.12–0.23) were the factors negatively associated to prenatal course attendance (Supplementary Table S4).

### Influenza and Pertussis Vaccine Receipt

With regards to vaccination, a higher pertussis vaccine uptake compared to influenza vaccine uptake was observed among all participants (52.3% vs. 21.5%, *p* < 0.001). We found a significantly higher vaccine coverage among immigrants compared to Italians only for influenza (influenza vaccine uptake: 39.5% vs. 19.8%, *p* < 0.0001; pertussis vaccine uptake: 59.7% vs. 51.6%, *p* = 0.10) (Table 2).

Among unvaccinated women, a significantly higher proportion of immigrant than Italian women stated the pandemic was the main reason for not receiving the influenza vaccine (23.2% vs. 13.5%, respectively; *p* = 0.04), as well as for not receiving the pertussis vaccine (37% vs. 18.6%, respectively; *p* = 0.005) (Table 2).

Study participants enrolled in Naples were less likely to receive influenza and pertussis vaccines compared with participants enrolled in Milan/Cesena (aOR = 0.26; 95% CI, 0.18–0.37 and aOR = 0.20; 95% CI, 0.15–0.26, respectively). Also, immigrants showed higher odds of being vaccinated against influenza compared to Italians (aOR = 1.78; 95% CI, 1.15–2.73) (Supplementary Table S4).

With regards to pertussis vaccination, women with medium education level showed less likelihood of being vaccinated compared with women with high education level. (aOR = 0.73; 95% CI, 0.56–0.96). Similarly, multiparous women were less likely to be vaccinated against pertussis than primiparous women (aOR = 0.76; 95% CI, 0.59–0.98) (Supplementary Table S4).

## Discussion

This multicenter study conducted in three tertiary hospitals in Italy highlights the divergence in prenatal care services access and utilization among two pregnant populations, immigrants versus Italian citizens during the COVID-19 pandemic. Even though the access to maternal health services are formally recognized healthcare rights to migrant women in Italy, irrespective of their legal or illegal resident status [12], differences in the use of ANC services between immigrant and Italian mothers have been well-documented before the pandemic [13]. A previous report using the data from the Certificates of Healthcare at Delivery (CeDAPs) showed that for the 3 indicators of frequency and adequacy of ANC assessed (first ANC visit, number of visits and number of scans during pregnancy), Italian women performed better than their immigrant counterparts [14].

During the pandemic, up to 12% maternal facilities ceased outpatient routine visits and almost 60% provided visits to a limited extent [8]. Nevertheless, in our study the average number of antenatal visits among Italian and immigrant women was high with 8 and 8.2 visits, respectively, in line with the current WHO recommendations [15]. However, a significantly higher percentage of Italian women underwent 8 or more antenatal visits compared to immigrant women. Interestingly, the ANC preferred setting differed significantly between Italian and immigrant women, with only 18% Italians compared to 45% immigrants who stated they received their antenatal visits in a hospital setting.

Similarly, although our results showed a high compliance for obstetric ultrasounds -at least 3- with 94% of global coverage and without significant differences between immigrants and Italians, the latter were more likely to use non-hospital settings. In addition, among women who did not perform an ultrasound exam, a higher proportion of immigrants than Italians stated that the COVID-19 pandemic was the main reason for non-compliance. According to the Italian CeDAP data, pregnant women who underwent more than 3 ultrasounds in Italy accounted for 71% and 74% of all live births in 2019 and 2020 [9, 16], respectively. The CeDAP figures and our findings indicate optimal ultrasound compliance for pregnant women during the pandemic. However, in our study we found that Italian women more frequently used non-hospital settings to receive this service.

In most European countries, public healthcare services provide the larger proportion of ANC, but in the case of Italy, a public–private partnership is in place [17]. Furthermore, previous evidence has revealed that Italian pregnant women used more private services than immigrant women [13]. During the pandemic, concerns about contracting the virus and reduction in availability of ANC appointments in the hospital could have led Italian women to choose even more frequently a private setting for their ANC services than during pre-pandemic years. In contrast, in the case of immigrant women, information about and access to a full range of options for prenatal care, out-of-pocket costs associated with choosing a private facility, as well as preferring to receive ANC and deliver in the same maternal facility could have prevented them from utilizing private settings as most Italian women did. The distinction between the sites for antenatal visits and obstetric scans preferred by immigrants and native-born women observed in our study might also be associated with the differences in socio-economic status and level of education between both groups. A significantly higher proportion of immigrants, in our study, had a lower level of education than Italians and more immigrant women, compared to autochthonous women, were housewives or unemployed. Finally, migrants might have had less access to public health messages related to the pandemic due to language barriers and weaker migrant social network ties compared to native-born women as it has been shown in pre-pandemic studies [18].

Our findings also showed that among Italian women, the ones enrolled in the Naples maternity hospital (Southern Italy) compared to the ones enrolled in the Milan or Cesena hospitals (Northern Italy) were more likely to have eight or more antenatal visits. This is consistent with CeDAP data that also showed regional differences in ANC services utilization with a higher number of antenatal visits and prenatal ultrasounds observed in Southern Italy compared to Northern Italy. For instance, 3 out of 5 women in Campania performed 7 ultrasounds or more compared to only 1 in 5 women in Lombardy and Emilia-Romagna regions during 2019 and 2020 [9, 16]. In addition, no differences were observed in the previous year versus the first year of the pandemic [9, 16].

With regards to Emergency visits, one prior single-center study reported a marked decrease in access to the Obstetric Emergency Department among Italians and no change was observed among immigrants during the pandemic [19]. Dell’Ultri et al enrolled women from February to June 2020 and they hypothesized that the media campaign reporting the struggle of the national health service might have influenced the seeking care behavior especially among Italian women, because most immigrant women had less access to media information due to language barriers [19]. In our study, Italian women reported more emergency visits than immigrant women. It is very likely that as the pandemic progressed the decreased utilization of Emergency care among Italian women observed by other authors during the initial pandemic phase returned to the pre-pandemic utilization patterns. Since we included women from May to December 2020, this might explain the difference between our findings and the results reported by Dell’Ultri et al. Additionally, the fact that immigrant mothers were younger and probably healthier than Italians in our study, similar to what has been observed in most Western countries [20], might have played a role in their lower demand for emergency care during the study period. Dell’Ultri et al. have also suggested that Italian women chose an environment considered safer, such as a private maternity practice, to avoid hospital access [19]. Our results confirm that Italian women chose the private settings more frequently than immigrants for most ANC services during the pandemic.

Among all the ANC services assessed during the first year of the pandemic, the prenatal course and maternal vaccinations showed the lowest compliance levels in our study. Overall, only around one-third of the study population attended a prenatal course, which is lower than the 44% figure reported in a previous report [21]. In addition, among women who did not attend the prenatal course, a significantly higher proportion of immigrants than Italians stated the COVID-19 pandemic was the main reason for non-compliance. Even though we did not identify significant differences in course attendance between Italians and Immigrants (33% vs. 38%), Italian women who did perform a prenatal course utilized the online version more frequently than Immigrant women (20% vs. 12%, respectively). The proportion of women aged 25 to 54 who used the internet in the last 3 months reached up to 86% in Italy [22]. However, since the migrant population has usually less stable employment conditions compared to the native population and migrants are overrepresented in a number of sectors such as the hospitality industry [23], it is very likely that immigrant women encountered more challenges to access telehealth services during the pandemic, such as the online version of prenatal courses. Similar groups, such as US refugees, showed having less access to telemedicine than non-refugees during the COVID-19 pandemic, mainly due to language barriers and other logistic issues [24]. Finally, women enrolled in Naples, with lower levels of education, housewives or unemployed as well as multiparous women were less likely to attend the prenatal course. These findings are in line with pre-pandemic studies showing that women with a high education level, living in Northern Italy and being primiparous were more likely to attend a prenatal course [25].

Regarding maternal immunization, we found a lower vaccine uptake of both influenza and pertussis vaccines among Italian women compared to immigrant women. However, only the influenza vaccine uptake was significantly higher in immigrants compared to native-born women. The lower pertussis vaccine coverage in Italian versus immigrant women observed in this study differed from a previous estimate reported by our research group during 2019 [26]. Interestingly, in the same study, we did not find an association between immigrant status and a higher influenza vaccine uptake [26], but in the present study immigrant mothers showed significantly higher coverage compared to autochthonous women. Moreover, regional differences in vaccine compliance were identified, with women enrolled in Naples showing the lowest maternal vaccine coverage. During the pandemic, the vaccine availability in the hospital setting might have represented an advantage for women receiving ANC visits in this type of facility and it might have positively impacted vaccine compliance. In the Milan study center, the vaccine was offered in the hospital, while in Naples, most pregnant patients were referred to the vaccine clinics to be vaccinated; this could partially explain the low maternal vaccine coverage found in this study center. Other factors, such as receiving a vaccine recommendation by a maternal care provider (MCP) has been shown to be the most important predictor to be vaccinated among pregnant women in Italy [26, 27]. However, during the pandemic, it is very likely that MCPs were focused on other priorities, and maternity care services faced significant barriers to providing certain prenatal care services including vaccinations. Finally, less extensive healthcare access among immigrants in Europe has been often attributed to their lower socio-economic status [28]. This situation might have been aggravated during the pandemic, rendering vaccination access even more difficult for non-native women.

Our study has some potential limitations. First, the rates of utilization of prenatal care services are based on self-reported practices provided by study participants. Second, since surveys are subject to selection bias, it might be possible that the proportion of immigrant women who accepted to participate differed from the target population. In our study sample, immigrant women represented 19%, 11% and 9.7% of all study participants enrolled in Milan (Lombardy region), Cesena (Emilia-Romagna region), and Naples (Campania region), respectively. In 2020, national data showed that births to immigrant mothers represented 30.4%, 32.3%, and 6.8% of all births in Lombardy, Emilia-Romagna and Campania, respectively [9]. Although the proportion of immigrant respondents is below the regional distribution, when we compared the make-up of immigrant mothers enrolled in our study with national figures, we observed a similar pattern. For instance, the most frequent foreign countries among our survey participants were Romania (15.8%), Morocco (11.4%), Albania (8.8%), China (7%), and Ukraine (6.1%). These results are similar to regional data in 2020, in which the top 3 foreign countries among all births in Italy were Romania, Morocco, and Egypt in Lombardy [29]; Morocco, Romania, and Albania in Emilia-Romagna [30], and Romania, Morocco, and Ukraine in Campania [31]. For this reason, we consider that our findings revealed valuable information about the disparities in maternity care access experienced during the COVID-19 pandemic by immigrant versus native-born women in Italy.

Our study also has several strengths. First, to our knowledge, this is the only Italian study which assessed the adequacy of prenatal care based on the utilization of 7 prenatal care services among Italian and immigrant women during the first year of the pandemic. Several studies have shown that COVID-19 pandemic has impacted negatively on maternal health outcomes along with maternity care provision worldwide [32]. However, scarce data is available about the indirect effects of the pandemic on vulnerable populations, such as immigrant pregnant women. Data prior to the pandemic, obtained from 10 European countries during 2018 and 2019, revealed that migrants in Italy and Austria scored higher in the Discrimination Scale to Medical Settings compared with other countries [33]. Additionally, the same study showed that migrant women had less access to healthcare services compared with migrant males [33]. Our findings highlight that pre-existing prenatal care organization, as well as the containment measures implemented during 2020 in Italy, might have exposed immigrant women to more challenges compared with Italian women when accessing prenatal care services.

Secondly, our study also revealed differences in utilization of prenatal care services by region. Italy has the third-largest immigrant population in Europe, after Germany and the United Kingdom [34] but the regional distribution of immigrants is not even across the country. For instance, in 2020, more than half (58%) of immigrants lived in Northern Italy (around 23% in Lombardy region and 11% in Emilia-Romagna region), while only 12% lived in Southern Italy (5% in Campania region) [35].

### Conclusion

According to our results, both Italian and immigrant women reported globally a good compliance to prenatal care services except for prenatal care course attendance and maternal vaccination. However, immigrants were more likely than Italians to attend hospital settings to receive prenatal care services and, among women not attending prenatal services, immigrants were more likely to state that the pandemic was the main reason for non-compliance. Our findings also showed significant regional differences in maternity care access in Italy during the COVID-19 pandemic probably related to an exacerbation of long-standing health inequities already existent in this country. In summary, the heterogeneous distribution of immigrant women in Italy requires that maternal care providers as well as policymakers have access to granular data to tailor maternity care services to the specific needs and barriers faced by immigrant women in each Italian region.
