# Supplementary material for: Impact of the COVID-19 Pandemic on Prenatal Care Utilization Among Italian and Immigrant Pregnant Women: A Multicenter Survey
Source: Int J Public Health. 2024 Feb 19;69:1606289. doi: 10.3389/ijph.2024.1606289 (PMC10910076; doi:10.3389/ijph.2024.1606289)
Supplement: Supplementary file 1 [file Table1.docx]

**Supplemental Table 1. Country of origin among immigrant women enrolled by study center in Italy from May to December 2020 (N=114).**

| Milan  (N= 64) | | | Cesena  (N= 39) | | | Naples  (N= 11) | | |
| --- | --- | --- | --- | --- | --- | --- | --- | --- |
|  | N | (%) |  | N | (%) |  | N | (%) |
| Romania | 8 | (12.5) | Albania | 9 | (23.1) | Ukraine | 4 | (36.4) |
| China | 7 | (10.9) | Romania | 8 | (20.5) | Romania | 2 | (18.2) |
| Morocco | 5 | (7.8) | Morocco | 7 | (17.9) | Other^c^ | 5 | (45.4) |
| Egypt | 4 | (6.2) | Polonia | 3 | (7.7) |  |  |  |
| Spain | 4 | (6.2) | Moldavia | 3 | (8.7) |  |  |  |
| Other^a^ | 36 | (56.3) | Other^b^ | 9 | (23.1) |  |  |  |
|  |  |  |  |  |  |  |  |  |

**Note:** Countries include into the *Other* category are specified below:

a= Brazil (3), Philippines (3), Peru (3), Russia (3), Ukraine (3), Belorussia (2), Bulgaria (2), El Salvador (2), Germany (2), Greece (2),

Albania (1), Argentine (1), Equator (1), France (1), Georgia (1), Honduras (1), Moldavia (1), Netherlands (1), Cech Republic (1),

Santo Domingo (1), US (1).

b= Bosnia (2), Bulgaria (2), Senegal (2), Spain (2), Brazil (1).

c= Cape Verde (1), China (1), Philippines (1), Morocco (1), Russia (1).
