# Supplementary material for: Impact of the COVID-19 Pandemic on Prenatal Care Utilization Among Italian and Immigrant Pregnant Women: A Multicenter Survey
Source: Int J Public Health. 2024 Feb 19;69:1606289. doi: 10.3389/ijph.2024.1606289 (PMC10910076; doi:10.3389/ijph.2024.1606289)
Supplement: Supplementary file 2 [file Table2.docx]

**Supplementary Table 2. Socio-demographic characteristics among Italian and immigrant women enrolled at the 3 study centers in Italy from May to December 2020 (N=1312).**

|  | **Italian women**  **(N= 1198)** | | | | | | | |  | **Immigrant women**  **(N= 114)** | | | | | | | |
| --- | --- | --- | --- | --- | --- | --- | --- | --- | --- | --- | --- | --- | --- | --- | --- | --- | --- |
|  | **Milan**  (N= 280) | |  | **Cesena**  (N= 318) | |  | **Naples**  (N= 600) | |  | **Milan**  (N= 64) | |  | **Cesena**  (N= 39) | |  | **Naples**  (N= 11) | |
|  | N | (%) |  | N | (%) |  | N | (%) |  | N | (%) |  | N | (%) |  | N | (%) |
| **Age group** |  |  |  |  |  |  |  |  |  |  |  |  |  |  |  |  |  |
| <35 | 144 | (51.43) |  | 210 | (66.04) |  | 392 | (65.33) |  | 48 | (75.00) |  | 26 | (66.67) |  | 8 | (72.73) |
| >=35 | 136 | (48.57) |  | 108 | (33.96) |  | 208 | (34.67) |  | 16 | (25.00) |  | 13 | (33.33) |  | 3 | (27.27) |
| **Marital status** |  |  |  |  |  |  |  |  |  |  |  |  |  |  |  |  |  |
| Married | 153 | (54.64) |  | 144 | (45.28) |  | 433 | (72.17) |  | 36 | (56.25) |  | 23 | (58.97) |  | 7 | (63.64) |
| Cohabiting/single | 127 | (45.36) |  | 174 | (54.72) |  | 167 | (27.83) |  | 28 | (43.75) |  | 16 | (41.03) |  | 4 | (36.36) |
| **Educational level^a^** |  |  |  |  |  |  |  |  |  |  |  |  |  |  |  |  |  |
| Low | 17 | (6.07) |  | 25 | (7.86) |  | 120 | (20) |  | 10 | (15.62) |  | 11 | (28.21) |  | 2 | (18.18) |
| Middle | 69 | (24.64) |  | 144 | (45.28) |  | 260 | (43.33) |  | 22 | (34.38) |  | 20 | (51.28) |  | 9 | (81.82) |
| High | 194 | (69.29) |  | 149 | (46.86) |  | 220 | (36.67) |  | 32 | (50.00) |  | 8 | (20.51) |  | 0 | (0.00) |
| **Work status** |  |  |  |  |  |  |  |  |  |  |  |  |  |  |  |  |  |
| Employed | 247 | (88.21) |  | 234 | (73.58) |  | 289 | (48.17) |  | 36 | (56.25) |  | 22 | (56.41) |  | 4 | (36.36) |
| Housewife/unemployed | 33 | (11.79) |  | 84 | (26.42) |  | 311 | (51.83) |  | 28 | (43.75) |  | 17 | (43.59) |  | 7 | (63.64) |
| **Parity** |  |  |  |  |  |  |  |  |  |  |  |  |  |  |  |  |  |
| Primiparous | 143 | (51.07) |  | 163 | (51.26) |  | 316 | (52.67) |  | 34 | (53.12) |  | 17 | (43.59) |  | 2 | (18.18) |
| Multiparous | 137 | (48.93) |  | 155 | (48.74) |  | 284 | (47.33) |  | 30 | (46.88) |  | 22 | (56.41) |  | 9 | (81.82) |
| **Pregnancy complications** |  |  |  |  |  |  |  |  |  |  |  |  |  |  |  |  |  |
| No | 243 | (86.79) |  | 275 | (86.48) |  | 513 | (85.50) |  | 50 | (78.12) |  | 38 | (97.44) |  | 9 | (81.82) |
| Yes | 37 | (13.21) |  | 43 | (13.52) |  | 87 | (14.50) |  | 14 | (21.88) |  | 1 | (2.56) |  | 2 | (18.18) |

^a^ Lower education = no secondary school diploma; Middle education = completed secondary school with diploma; Higher education = continued education beyond secondary school.
