# Supplementary material for: Impact of the COVID-19 Pandemic on Prenatal Care Utilization Among Italian and Immigrant Pregnant Women: A Multicenter Survey
Source: Int J Public Health. 2024 Feb 19;69:1606289. doi: 10.3389/ijph.2024.1606289 (PMC10910076; doi:10.3389/ijph.2024.1606289)
Supplement: Supplementary file 3 [file Table3.docx]

| **Variables.** | **8 or more prenatal care visits** | | **Emergency Room Visit** | | **Flu vaccination** | | **Pertussis Vaccination** | | **Prenatal Course Attendance** | |
| --- | --- | --- | --- | --- | --- | --- | --- | --- | --- | --- |
|  | Compliance  N (%) | Unadjusted  OR (95% CI) | Compliance  N (%) | Unadjusted  OR (95% CI) | Compliance  N (%) | Unadjusted  OR (95% CI) | Compliance  N (%) | Unadjusted  OR (95% CI) | Compliance  N (%) | Unadjusted  OR (95% CI) |
| City  Milan/Cesena  Naples | 397 (56.6)  436 (71.4) | ref.  1.91 (1.52-2.45) | 380 (54.2)  489 (80.0) | ref.  3.39 (2.64-4.35) | 217 (30.9)  65 (10.6) | ref.  0.27 (0.19-0.36) | 491 (70.0)  195 (31.9) | ref.  0.20 (0.16-0.25) | 328 (46.8)  112 (18.3) | ref.  0.26 (0.20-0.33) |
| Citizenship  Italian  Immigrant | 771 (64.4)  62 (54.4) | ref.  0.66 (0.45-0.98) | 807 (67.4)  62 (54.4) | ref.  0.57 (0.39-0.85) | 237 (19.8)  45 (39.5) | ref.  2.64 (1.76-3.94) | 618 (51.6)  68 (59.6) | ref.  1.39 (0.94-2.06) | 397 (33.1)  43 (37.7) | ref.  1.22 (0.82-1.81) |
| Maternal age  < 35 years  ≥35 years | 530 (64.0)  303 (62.6) | ref.  0.94 (0.75-1.19) | 564 (68.1)  305 (63.0) | ref.  0.80 (0.63-1.01) | 175 (21.1)  107 (22.1) | ref.  1.06 (0.81-1.39) | 428 (51.7)  258 (53.3) | ref.  1.07 (0.85-1.34) | 278 (33.6)  162 (33.5) | ref.  1.00 (0.78-1.26) |
| Education Level  High  Middle  Low | 333 (63.6)  376 (62.4)  124 (67.0) | ref.  1.05 (0.83-1.34)  1.23 (0.87-1.75) | 375 (62.2)  361 (68.9)  133 (71.9) | ref.  1.56 (1.09-2.24)  1.35 (1.05-1.73) | 152 (25.2)  98 (18.7)  32 (17.3) | ref.  0.68 (0.51-0.91)  0.62 (0.40-0.94) | 368 (61.0)  245 (46.8)  73 (39.5) | ref.  0.56 (0.44-0.71)  0.42 (0.30-0.58) | 281 (46.6)  128 (24.4)  31 (16.8) | ref.  0.37 (0.29-0.48)  0.23 (0.15-0.35) |
| Civil status  Married  Single/ Cohabiting | 515 (64.7)  318(61.6) | ref.  0.88 (0.70-1.10) | 523 (65.7)  346 (67.1) | ref.  1.06 (0.84-1.34)) | 168 (21.1)  114(22.1) | ref.  1.06 (0.81-1.39) | 396 (49.6)  290 (56.2) | ref.  1.30 (1.04-1.62) | 236 (29.7)  204 (39.5) | ref.  1.55 (1.23-1.96) |
| Employment status  Employed  Housewife/ Unemployed | 538 (64.6)  295 (61.5) | ref.  0.87 (0.69-1.10) | 522 (62.7) 347 (72.3) | ref.  1.55 (1.22-1.98) | 193 (23.2)  89 (18.5) | ref.  0.75 (0.57-0.99) | 493 (59.3)  193 (40.2) | ref.  0.46 (0.37-0.58) | 352 (42.3)  88 (18.3) | ref. 0.31 (0.23-0.40) |
| Parity  Primipara  Multipara | 446 (66.1)  387 (60.8) | ref.  0.79 (0.63-1.00) | 477 (70.7)  392 (61.5) | ref.  0.66 (0.53-0.84) | 155 (23.0)  127 (19.9) | ref.  0.84 (0.64-1.09) | 376 (55.7)  310 (48.7) | ref.  0.75 (0.61-0.94) | 333 (49.3)  107 (16.8) | ref.  0.21 (0.16-0.27) |
| Pregnancy Complications  No  Yes | 695 (61.6)  138 (75.0) | ref.  1.87(1.32-2.69) | 742 (65.8)  127 (69.0) | ref.  1.16 (0.83-1.63)) | 250 (22.2)  32 (17.4) | ref.  0.74 (0.49-1.10) | 601 (53.3)  85 (46.2) | ref.  0.75 (0.55-1.03) | 383 (34.0)  57 (31.0) | ref.  0.87 (0.62-1.22) |
| Number of ANC visits  <8 visits  >=8 visits | ---  --- | ---  --- | 308 (64.3)  561 (67.4) | ref.  1.15 (0.90-1.45) | 108 (22.6)  174 (20.9) | ref.  0.91 (0.69-1.19) | 256 (53.4)  430 (51.6) | ref.  0.93 (0.74-1.16) | 166 (34.7)  274 (32.9) | ref.  0.92 (0.73-1.17) |
