# Supplementary material for: Impact of the COVID-19 Pandemic on Prenatal Care Utilization Among Italian and Immigrant Pregnant Women: A Multicenter Survey
Source: Int J Public Health. 2024 Feb 19;69:1606289. doi: 10.3389/ijph.2024.1606289 (PMC10910076; doi:10.3389/ijph.2024.1606289)
Supplement: Supplementary file 4 [file Table4.docx]

| **Variables.** | **8 or more prenatal care visits^a^** | | **Emergency Room Visit^b^** | | **Flu vaccination^b^** | | **Pertussis Vaccination^b^** | | **Prenatal Course Attendance^b^** | |
| --- | --- | --- | --- | --- | --- | --- | --- | --- | --- | --- |
|  | Compliance  N (%) | Adjusted  OR (95% CI) | Compliance  N (%) | Adjusted  OR (95% CI) | Compliance  N (%) | Adjusted  OR (95% CI) | Compliance  N (%) | Adjusted  OR (95% CI) | Compliance  N (%) | Adjusted  OR (95% CI) |
| City  Milan/Cesena  Naples | 397 (56.6)  436 (71.4) | ref.  **2.03 (1.56-2.65)^***^** | 380 (54.2)  489 (80.0) | ref.  **3.40 (2.57-4.54)^***^** | 217 (30.9)  65 (10.6) | ref.  **0.26 (0.18-0.37)^***^** | 491 (70.0)  195 (31.9) | ref.  **0.20 (0.15-0.26)** | 328 (46.8)  112 (18.3) | ref.  **0.24 (0.17-0.32)^***^** |
| Citizenship  Italian  Immigrant | 771 (64.4)  62 (54.4) | ref.  0.88 (0.59-1.33) | 807 (67.4)  62 (54.4) | ref.  0.87 (0.58-1.32) | 237 (19.8)  45 (39.5) | ref.  **1.78 (1.15-2.73)^**^** | 618 (51.6)  68 (59.6) | ref.  0.78 (0.51-1.22) | 397 (33.1)  43 (37.7) | ref.  0.95 (0.59-1.53) |
| Maternal age  < 35 years  ≥35 years | 530 (64.0)  303 (62.6) | ref.  0.97 (0.75-1.24) | 564 (68.1)  305 (63.0) | ref.  0.93 (0.72-1.21) | 175 (21.1)  107 (22.1) | ref.  1.09 (0.81-1.46) | 428 (51.7)  258 (53.3) | ref.  0.99 (0.76-1.27) | 278 (33.6)  162 (33.5) | ref.  1.22 (0.92-1.64) |
| Education Level  High  Middle  Low | 333 (63.6) 376 (62.4)  124 (67.0) | ref.  1.11 (0.85-1.46)  1.31 (0.88-1.94) | 375 (62.2)  361 (68.9)  133 (71.9) | ref.  1.12 (0.85-1.49)  1.11 (0.73-1.69) | 152 (25.2)  98 (18.7)  32 (17.3) | ref.  0.79 (0.57-1.08)  0.82 (0.50-1.32) | 368 (61.0)  245 (46.8)  73 (39.5) | ref.  **0.73 (0.56-0.96)^*^**  0.72 (0.48-1.07) | 281 (46.6)  128 (24.4)  31 (16.8) | ref.  **0.48 (0.35-0.65)^***^**  **0.45 (0.27-0.73)^***^** |
| Civil status  Married  Single/ Cohabiting | 515 (64.7)  318(61.6) | ref.  0.94 (0.73-1.21) | 523 (65.7)  346 (67.1) | ref.  1.26 (0.97-1.65) | 168 (21.1)  114(22.1) | ref.  0.80 (0.59-1.08) | 396 (49.6)  290 (56.2) | ref.  0.92 (0.71-1.20) | 236 (29.7)  204 (39.5) | ref.  1.01 (0.75-1.35) |
| Employment status  Employed  Housewife/ Unemployed | 538 (64.6)  295 (61.5) | ref.  **0.65 (0.49-0.86)^***^** | 522 (62.7) 347 (72.3) | ref.  1.09 (0.82-1.46) | 193 (23.2)  89 (18.5) | ref.  1.25 (0.89-1.74) | 493 (59.3)  193 (40.2) | ref.  0.86 (0.65-1.14) | 352 (42.3)  88 (18.3) | ref. **0.65 (0.47-0.89)^**^** |
| Parity  Primipara  Multipara | 446 (66.1)  387 (60.8) | ref.  0.80 (0.62-1.02) | 477 (70.7)  392 (61.5) | ref.  **0.66 (0.51-0.86)^***^** | 155 (23.0)  127 (19.9) | ref.  0.77 (0.57-1.03) | 376 (55.7)  310 (48.7) | ref.  **0.76 (0.59-0.98)^*^** | 333 (49.3)  107 (16.8) | ref.  **0.17 (0.12-0.23)^***^** |
| Pregnancy Complications  No  Yes | 695 (61.6)  138 (75.0) | ref.  **1.97(1.38-2.85)^***^** | 742 (65.8)  127 (69.0) | ref.  1.18 (0.83-1.70) | 250 (22.2)  32 (17.4) | ref.  0.74 (0.48-1.13) | 601 (53.3)  85 (46.2) | ref.  0.76 (0.54-1.07) | 383 (34.0)  57 (31.0) | ref.  1.10 (0.74-1.62) |
| Number of ANC visits  <8 visits  >=8 visits | ---  --- | ---  --- | 308 (64.3)  561 (67.4) | ref.  0.92 (0.72-1.19) | 108 (22.6)  174 (20.9) | ref.  1.15 (0.86-1.54) | 256 (53.4)  430 (51.6) | ref.  1.21 (0.94-1.56) | 166 (34.7)  274 (32.9) | ref.  1.04 (0.74-1.62) |

^a The variables include in this model are: city, citizenship, maternal age, education level, civil status, employment status, parity and comorbidity.^

^b The variables include in each one of these models are: city, citizenship, maternal age, education level, civil status, employment status, parity, comorbidity and number of ANC visits.^

^Note: ***: p<0.001; **: p<0.01; *: p<0.05^
